# Targeting AMP-Activated Protein Kinase in Aging-Related Cardiovascular Diseases

**DOI:** 10.14336/AD.2019.0901

**Published:** 2020-07-23

**Authors:** Tian Li, Nan Mu, Yue Yin, Lu Yu, Heng Ma

**Affiliations:** ^1^Department of physiology and pathophysiology, School of Basic Medicine, Fourth Military Medical University, Xi'an, China; ^2^Department of pathology, Xijing Hospital, Fourth Military Medical University, Xi’an, China

**Keywords:** AMPK, Aging, Cardiovascular diseases, Cardioprotecion

## Abstract

Aging is a pivotal risk factor for developing cardiovascular diseases (CVD) due to the lifelong exposure to various risk factors that may affect the heart and vasculature during aging. AMP-activated protein kinase (AMPK), a serine/threonine protein kinase, is a pivotal endogenous energy regulator that protects against various pathological alterations. In this report, we first introduced the protective mechanisms of AMPK signaling in myocardium, such as oxidative stress, apoptosis, inflammation, autophagy and inflammatory response. Next, we introduced the potential correlation between AMPK and cardiac aging. Then, we highlighted the roles of AMPK signaling in cardiovascular diseases, including myocardial ischemia, cardiomyopathy, and heart failure. Lastly, some potential directions and further perspectives were expanded. The information extends our understanding on the protective roles of AMPK in myocardial aging, which may contribute to the design of drug targets and sheds light on potential treatments of AMPK for aging-related CVD.

## 1. Introduction

Over the past decades, the human life expectancy has been greatly prolonged due to the improvement of medical and health conditions leading to a high prevalence of aging-related diseases, such as cardiovascular diseases (CVD), cerebrovascular disease, cancer, et al [1]. According to the Heart Disease and Stroke Statistics 2019, it is reported that CVD causes immense health and economic burdens in the United States and globally [2]. Burden and mortality of most CVD can be traced back to four adverse health behaviors (smoking, poor diet, elevated body mass index, sedentary lifestyle) and three major risk factors (hypercholesterolemia, hypertension, diabetes) [3].

Myocardial contractile function displays a dramatic decline with aging and contributes to the cardiac morbidity and mortality in the elderly [4]. Surprisingly, although there have been advances in care that have spurred improvements in cardiovascular outcomes, cardiovascular disease remains the leading cause of death around the world [5]. In 1995, it was found that AMP-activated protein kinase (AMPK) was activated during myocardial ischemic reperfusion (MIR), which is associated with high rates of fatty acid oxidation [6]. Before long, Sambandam et al. found that AMPK promotes glucose uptake, glycolysis, and fatty acid oxidation during MIR [7]. Previous studies have suggested that dysfunctional metabolism involving glucose and fatty acid (FA) is closely related to myocardial ischemic injury. Accumulating evidence has suggested that the activation of AMPK may confer cardioprotection in aging heart, which is the pathological alteration and terminal stage of CVD. AMPK, which is widely distributed in various cells and organs as a heterotrimeric protein complex, consists of 3 subunits including catalytic α (66kDa) subunits, regulatory β (38 kDa) and γ (38 kDa) subunits which occur as multiple isoforms (α1/α 2; β1/β2; γ1/γ2/γ3) encoded by distinct genes [8]. Compelling evidence has indicated that activated AMPK ameliorates myocardial ischemia-reperfusion injury (IRI) [8-10]. while the absence of AMPK may promote IRI in an aged heart [11].

This review focuses on the roles of AMPK in aging-related CVD. First, we introduce protective mechanisms of AMPK signaling in myocardium and cardiac aging. Thereafter, we highlight the roles of AMPK in CVD. Eventually, therapeutic targets and possible mechanisms are described. In summary, the information compiled here will serve as a helpful reference for AMPK and hopefully aid in the design of future experimental studies and therapeutic target in aging-related CVD.

## 2. Protective mechanisms of AMPK signaling in myocardium

### 2.1 Oxidative stress

Oxidative stress refers to the pathological imbalance between the production of reactive oxygen species (ROS) and the endogenous antioxidant defense system [12]. Excessive ROS has a negative effect on the myocardial calcium handling, arrhythmia, and cardiac remodeling as it induces hypertrophic signaling, apoptosis, and necrosis [13]. Advances in cardiovascular research have identified oxidative stress as an important pathophysiological pathway in the development and progression of cardiac aging and heart failure.

Some studies have uncovered some mechanisms involving oxidative stress and AMPK in myocardium. Recently, there is evidence to support that a series of hormones and natural drugs are implicated in AMPK activation. Fibroblast growth factor (FGF) 19, an endocrine-derived hormone, can induce the activation of AMPK under hyperglycemia. Li et al used streptozotocin (STZ)-induced diabetic mice model and observed that FGF19 treatment leads to a significant increase in Nrf2 and HO-1 protein expression. However, treatment with AMPK inhibitor compound C remarkably reverses the above-mentioned effects, suggesting that FGF19 has a protective role on the heart by averting oxidative stress-related diabetic cardiomyopathy through activation of the AMPK/Nrf2/HO-1 pathway [14]. In addition, Yang’s group found that FGF21 has physiological and pharmacological functions that prevent type 2 diabetic lipotoxicity-induced cardiomyopathy through activation of both AMPK/Akt2/Nrf2-mediated antioxidative pathway and AMPK/ACC/CPT-1-mediated lipid-lowering effect [15]. Fortunellin is a natural plant product isolated from the fruits of Fortunella margarita and belongs to flavonoids. Zhao et al observed that Fortunellin can recover high fructose-induced low levels of p-AMPK *in vivo* and *in vitro*. They found that Fortunellin protected against fructose-induced inflammation and oxidative stress by enhancing AMPK/Nrf2/HO-1 pathway in diabetic mice and cardiomyocytes with fructose treatment [16]. Kosuru and colleagues discovered that pterostilbene significantly reduces post-ischemic cardiac infarct size, hypoxia/reoxygenation-induced oxidative stress, and increases the activity of AMPK in diabetic rats cardiomyocytes [17]. In Kosuru’s subsequent studies, it revealed that the effect above is achieved by activating AMPK/Nrf2/HO-1 signaling [18]. Guo’s group demonstrated that resveratrol protects cardiomyocytes against high glucose-induced apoptosis through suppression of NADPH oxidase-derived ROS generation and maintenance of endogenous antioxidant defenses in primary cultured neonatal rat cardiomyocytes, which are mostly mediated by AMPK related pathway [19]. Taken together, these findings suggest that AMPK protects against oxidative stress in the heart and acts as a potential therapeutic target.

### 2.2 Apoptosis

Apoptosis is the process of programmed cell death, which is considered as a vital component of cell life including normal cell turnover, proper development, and functioning of the immune system, hormone-dependent atrophy et al [20, 21]. Numerous studies have recently reported that inappropriate apoptosis (either too little or too much) is a factor involved in various diseases including neurodegenerative diseases, CVD, and cancer [22-25].

Previously, it has been reported that the inhibition of AMPK results in the initiation of cardiac apoptosis, suggesting that the regulation of AMPK pathway has an important function in the cardiovascular system (CVS) [26, 27]. Current studies have confirmed that endoplasmic reticulum (ER) stress (ERS) is associated with cardiomyocyte apoptosis [28, 29]. Under diverse stress conditions such as ischemia, the normal function of the ER becomes impaired leading to an imbalance in the protein homeostasis networks with concomitant accumulation of misfolded proteins in the ER lumen, called ERS [30]. Notably, chronic and persistent ERS may induce the degenerative ERS pathway, which leads to apoptosis. Nam et al discovered that activated protein C (APC) protects against methylglyoxal (MGO)-induced myocyte apoptosis via inhibiting ERS [31]. They demonstrated that APC can activate AMPK signaling which has been shown to prevent ER stress-induced apoptosis [32]. Melatonin, a documented potent antioxidant, can markedly alleviate the doxorubicin (DOX)-induced acute cardiac dysfunction via AMPK/PGC1α signaling pathway. Liu and colleagues found melatonin cotreatment could markedly inhibit acute DOX-induced apoptosis by decreasing the levels of pro-apoptotic protein (Bax and caspase 3) and elevate the levels of anti-apoptotic Bcl2 protein both *in vivo* and *in vitro*. Even so, the inhibition of both AMPK and PGC1α partially reversed the above effects exerted by melatonin, suggesting that the AMPK/PGC1α pathway is involved in the anti-apoptotic protective effects of melatonin against DOX cardiotoxicity [33]. In addition, Al-Damry’s group demonstrated that sitagliptin protected against cardiac apoptosis by activating LKB/AMPK/Akt pathway as well as reducing the activity of GSK3β and p38α/MAPK [34].

### 2.3 Autophagy

Autophagy is a life process of lysosome-mediated misfolded protein and aging damaged organelle degradation that plays a crucial role in the maintenance of cellular homeostasis and the recycling of material and energy [35-37]. Under physiological conditions, the level of autophagy is low but is rapidly increased when stimulated by various injury factors in the internal and external environments [35]. Compelling evidence has indicated that autophagy has major role in the regulation of cardiac homeostasis and function [36, 38].

An increasing body of evidence has uncovered that some mechanisms are involved in AMPK and autophagy in the heart. He et al demonstrated that AMPK restores cardiac autophagy and prevents cardiomyopathy in diabetic mice and high glucose-treated H9c2 cells. Exposure of H9c2 cells to high glucose reduces AMPK activity, inhibits Jun NH2-terminal kinase 1 (JNK1)-B-cell lymphoma 2 (Bcl-2) signaling, and promotes Beclin1 binding to Bcl-2. Nevertheless, the activation of AMPK by metformin stimulates JNK1/Bcl-2 signaling and dissociates Beclin1 and Bcl-2, thus restoring cardiac autophagy. Therefore, the dissociation of Bcl2 from Beclin1 may be a significant mechanism by which AMPK activation restores autophagy, protects against cardiac apoptosis, and prevents diabetic cardiomyopathy [39]. This was also in good agreement with another report [40]. Irisin, a novel polypeptide hormone proteolytically processed from fibronectin type III domain-containing protein 5 (FNDC5), protects against cardiac hypertrophy and improves chronic pressure overload-impaired autophagy flux via the activation of AMPK signaling [41]. Previous researches have shown that AMPK activates autophagy by two different pathways: deactivating mTORC1 or directly phosphorylating a protein kinase, ULK1 [42]. Li and colleagues discovered irisin activates autophagy via AMPK/ULK1 signaling independent of mTOR-S6K. Whereas autophagy may be protective during ischemia, it may be detrimental during reperfusion [43, 44]. Wang et al found that metformin could restore autophagy flux during MI/R via promoting both cytoplasmic AMPKα1- and nuclear AMPKα2-related signaling [45]. They observed, that during the reperfusion period, AMPKα1/mTOR/TFEB and AMPKα2/Skp2/ CARM1/TFEB signaling was inhibited, which contributed to autophagy suppression. Particularly, metformin can reverse the above effects. This suggests that increasing the TFEB level by AMPK may restore the cardiac function during MI/R. Recently, Li et al identified the correlation between nuclear AMPK and CARM1 in aging heart [46]. In aged heart, AMPK deficiency increased the expression of S-phase kinase-associated protein 2 (SKP2), which is responsible for degrading CARM1, eventually contributing to autophagy dysfunction in aged hearts. Notably, Ren et al discovered that Akt2-AMPK double knockout mice. Besides, Akt2-AMPK double knockout restrains autophagy by decreasing Atg5, Atg7, Beclin1, LC3BII/LC3BI ratio and increased p62; and suppresses mitophagy by reducing Parkin, Pink1, Bnip3, FundC1, PGC-1α, UCP2, and TFEB. The data indicates that Akt2-AMPK double ablation predisposes cardiac aging possibly related to compromised autophagy and mitophagy [47]. Moreover, they also discovered that Akt2 ablation prolongs life span and improves myocardial contractile function with adaptive cardiac remodeling via SIRT1-mediated autophagy regulation [48], whereas chronic Akt activation accentuates aging-induced cardiac hypertrophy and myocardial contractile dysfunction [49]. ALDH2 enzyme may suppress myocardial autophagy possibly through IKKβ-/AMPK-dependent mechanism en route to accentuation of myocardial remodeling and contractile dysfunction in aging [50]. Thus, the mechanisms of autophagy during myocardial reperfusion should be the focus of new research. Taken together, these findings demonstrate that AMPK-dependent autophagy plays a protective role in the heart during ischemia.

### 2.4 Inflammation

Inflammation is a defensive response that underlies a wide variety of physiological and pathological processes [51, 52]. It is worth noting that chronic inflammation has a decisive role in the development of numerous diseases such as diabetes, fatty liver disease, CVD and so on [53]. Current studies have uncovered some mechanisms involving AMPK and inflammatory response in the heart [16, 54].

Studies have found that inflammation in the early stages of infarction may not be entirely detrimental, it may even exert some salutary effects by promoting autophagy. However, at 4 weeks after left coronary artery (LCA) ligation, the levels of inflammatory cytokines are still substantially higher in the heart, suggesting a heightened state of inflammation in the chronically ischemic heart [55]. AMPK activation significantly attenuates the JNK/NF-κB signaling cascade and inhibits mRNA and protein levels of pro-inflammatory cytokines, such as TNF-α and IL-6, during hypoxia/reoxygenation in H9c2 cells, suggesting that AMPK may play a protective role in early myocardial ischemia [54]. Interestingly, Ren et al found that autophagy was dampened through an AMPK/mTOR/ULK1-dependent mechanism in the adiponectin deficiency, which exacerbates the cardiac dysfunction, apoptosis, and inflammation [56]. In addition, administration of PT significantly decreases cardiac hypertrophy, hypertension, oxidative stress, inflammation, NF-κB expression, and NLRP3 inflammasome in fructose-fed rats [18]. Wang et al administered TNF-α inhibitor to the mice daily during the first week of myocardial infarction and discovered that the TNF-α inhibitor remarkably inhibited autophagy and promoted myocyte apoptosis in the border zone [55]. Furthmore, Cieslik and colleagues found that AICAR treatment suppresses excessive monocyte chemoattractant protein-1 (MCP-1) generation, which diminished leukocyte infiltration and in consequence inhibits the formation of macrophage-derived myeloid fibroblasts. Interestingly, the number of mesenchymal fibroblasts is also reduced. Mechanically, AICAR-mediated AMPK activation inhibits the deposition of ECM and fibrosis in aging heart [57]. Aging promotes cardiac inflammation, the effect was attenuated by migration inhibitory factor (MIF) knockout. Intriguingly, aging-induced unfavorable responses were reversed by treatment with the autophagy inducer rapamycin, with improved myocardial ATP availability in aged WT and MIF^-/-^ mice. Using an in vitro model of senescence, MIF knockdown exacerbates doxorubicin-induced premature senescence in H9c2 myoblasts, the effect was ablates by MIF replenishment, possibly via activated AMPK [58]. Therefore, early treatment with anti-inflammatory drug may adversely affect the cardiac function. Therefore, we can deduce that AMPK may play a pivotal role in early MI by anti-inflammation, promote autophagy and anti-apoptosis.

The interplay of the above mechanisms and pathways contribute to cardioprotection of AMPK. AMPK activation has pleiotropic effects in the heart and which mechanisms are essential to the cardioprotective actions of AMPK in aging heart remain to be further explored (Table 1).

**Table 1:** Protective mechanisms of AMPK signaling in myocardium.

Models	Regulation	Effects	References
**C57BL/6 mice**	Oxidative stress	FGF19 activates AMPK to prevent ROS production protect the diabetic cardiomyocytes from oxidative stress induced damage	Li et al. (2018) [14]
**H9C2 cells / Diabetic mice**	Oxidative stress and Inflammation	Fortunellin protects against fructose-induced inflammation and oxidative stress by enhancing AMPK/Nrf2 pathway	Zhao et al. (2017) [16]
**Sprague-Dawley rats**	Oxidative stress and Apoptosis	Pterostilbene activates AMPK to suppress cardiac oxidative stress and apoptosis	Kosuru et al. (2018) [17]
**Neonatal rat cardiomyocytes**	Oxidative stress	Resveratrol inhibits ROS production by increasing phosphorylation of AMPK	Guo et al. (2015) [19]
**H9C2 cells/C57BL/6 mice**	Oxidative stress and Apoptosis	Melatonin inhibits apoptosis and oxidative stress via the activation of AMPK and upregulation of PGC1α with its downstream signaling	Liu et al. (2018) [33]
**H9c2 cells**	Autophagy	AMPK restores autophagy and protects against cardiac apoptosis.	He et al. (2013) [39]
**FNDC5(irisin-precursor) homozygous knockout (FNDC5-KO) mice / Cardiomyocytes from neonatal Sprague-Dawley rats**	Autophagy	Irisin protects against cardiac hypertrophy by inducing protective autophagy and autophagy flux via activating AMPK-ULK1 signaling	Li et al. (2018) [41]
**APN knockout (APNKO) mice**	Apoptosis and Inflammation	Adiponectin knockout exacerbates LPS-induced cardiac dysfunction	Ren et al. (2016) [56]
**H9c2 cells**	Inflammation	AMPK activation inhibits mRNA and protein levels of pro-inflammatory cytokines, such as TNF-α and IL-6	Chen et al. (2018) [54]

## 3. AMPK and cardiac aging

Aging is the most important risk factor that results in the development of CVD because of the lifelong exposure to various cardiovascular risk factors and specific environmental alterations affecting the heart and the vasculature during aging [59-62]. Cardiac aging manifests as a decline in function leading to heart failure. Vascular aging, a major risk factor for CVD, refers to the structural and functional defects that occur in the aorta during the aging process [63]. It’s important to consider that aging and heart disease are the main health-care burdens of industrialized nations in the 21st century, therefore it is particularly important to find effective therapies to restore the function of aging heart. One study reported that aging affects the cardioprotective AMPK signaling pathway, which plays a pivotal role in the increased susceptibility to myocardial ischemia observed in older cardiac patients [64]. Recently, compelling evidence has indicated that AMPK is a potential target that improves the function of aging heart and delay the aging process [46, 65].

Accumulating evidence suggests that the activation of AMPK signaling pathway can significantly reduce myocardial injury in aging hearts. Li et al found that AMPK activation is markedly impaired in aged hearts following IR compared to young hearts [65]. They further found that cardiac SIRT1 mediates AMPK activation via LKB1(Liver kinase B1, LKB1) deacetylation, and AMPK modulates SIRT1 activity via regulation of NAD^+^ level during MI. Importantly, the SIRT1 protein level and activity decline in aging heart, resulting in the impairment of the AMPK signaling. This suggests that SIRT1 and AMPK agonists have therapeutic potential for treatment of aging-related ischemic heart disease [65]. It has been shown that autophagy plays an active role in MI. Nonetheless, autophagy downregulation leads to cardiomyocyte dysfunction in aged hearts compared to young hearts [46, 66]. Li and colleagues observed that fasting induces AMPK-FoxO3a phosphorylation is significantly reduced in the aged heart which leads to the decline of CARM1 and impairment of the autophagy flux [46]. Surprisingly, AMPK dependent FoxO3a phosphorylation increases in the nucleus after AMPK activator AICAR treatment which results in the reduction of SKP2 and increase of CARM1. Finally, autophagy rejuvenates in senescent hearts. This indicates that the restoration of autophagy via activation of AMPK signaling can restore the cardiac function and decrease symptoms in an aged heart. These findings suggest that AMPK may be a potential strategy for delaying cardiac aging.

## 4. AMPK and aging-related CVD

### 4.1 Myocardial ischemia

Myocardial ischemia is the early pathological stage of CVD, such as myocardial infarction, and heart failure [67]. Incidence of myocardial IR injury is rapidly increasing worldwide. To limit the myocardial infarct size and reduce mortality, early restoration of blood flow to the ischemic myocardium is a common treatment strategy.

An increasing body of evidence indicates that AMPK has a major role in myocardial ischemia. Omentin, an adipokine, ameliorates acute ischemic injury in the heart by suppressing myocyte apoptosis via both AMPK- and Akt-dependent mechanisms [68]. Kataoka et al found that systemic administration of human omentin to mice reduced the myocardial infarct size and apoptosis after I/R, but the effects were reversed after inhibition of AMPK and Akt activity. *In vitro*, omentin suppresses apoptosis after hypoxia/reoxygenation, which is blocked by inactivation of AMPK or Akt. These results suggest that AMPK can protect heart against I/R injury *in vivo* or *in vitro*. As we all know, oxidative stress can aggravate the progression of myocardial ischemia. Trimetazidine, anti-anginal drug, significantly activates AMPK signaling during reperfusion, leading to the reduction of oxidative stress in the I/R hearts [69]. It is important to note that mitochondrial energy metabolism and oxidative stress play a crucial role in ameliorating myocardial ischemia/reperfusion injury. Tian et al. found that tilianin significantly reduces myocardial infarction, improves the pathological morphology of myocardium, markedly increase the contents of ATP and NAD+, decreases ADP and AMP contents and the ratio of AMP/ATP, reduce the level of ROS and MDA, enhance SOD activity, evidently increase the levels of AMPK, SIRT1 and PGC-1α mRNA, up-regulate the expressions of AMPK, pAMPK, SIRT1, PGC-1α, NRF1, TFAM and FOXO1 proteins. Interestingly, the above effect can be reserved by Compound C (a specific AMPK inhibitor) and EX-527 (a specific SIRT1 inhibitor) [70]. These results indicate that tilianin could attenuate MIRI by improving mitochondrial energy metabolism and reducing oxidative stress via AMPK/SIRT1/PGC-1α signaling pathway. Recently, Zhang’s group demonstrated that melatonin promotes the AMPK-OPA1-mitochondrial fusion/mitophagy axis against cardiac IR injury, which is also closely related to mitochondria [71]. Moreover, Sestrins are conserved proteins that accumulate in cells exposed to stress and potentiate adenosine monophosphate-activated protein kinase (AMPK) and inhibit activation of target of rapamycin (TOR) [72]. C57BL/6 mice were subjected to left anterior descending coronary artery occlusion for regional ischemia. Quan and colleguaes found that the protein level of Sesn2 in hearts was gradually decreased with aging. Notably, ischemic AMPK activation was attenuated in aged hearts compared with young hearts. The AMPK downstream glucose uptake and the rate of glucose oxidation were significantly impaired in aged hearts during ischemia and reperfusion. Furthermore, Sesn2 knockout hearts show a cardiac phenotype and response to ischemic stress that is similar to wild-type aged hearts [73]. Taken together, the activation of AMPK may be an ideal therapeutic target in MIRI.

### 4.2 Cardiomyopathy

Cardiomyopathies are a heterogeneous group of heart muscle diseases and an important cause of heart failure (HF). Compelling evidence has indicated that AMPK may play a pivotal role in cardiomyopathy [15, 74].

In 2015, the first mouse model of AMPK deficiency demonstrated that β1β2M-KO mice suffered from dilated cardiomyopathy [75]. This indicate that AMPK can be a potential strategy to treat various cardiomyopathies. Mulberry granules (MLD), a traditional Chinese medicine prescription, protects against diabetic-associated cardiomyopathy via the AMPK/Nrf2 signaling pathway [74]. By studying an experimental myocardial ischemia/reperfusion (MI/R) injury model in diabetes rats, Liu et al found that the pretreatment with MLD significantly induces the expression of Nrf2 and also increases AMPK signaling which is upstream and is required for Nrf2 activation. The results suggested that MLD may suppress oxidative stress induced by hyperglycemia and MI/R by activating AMPK/Nrf2 signaling pathway, and eventually protect against diabetic-associated cardiomyopathy. Al-Damry’s group demonstrated that sitagliptin protects against diabetic cardiomyopathy by attenuating apoptosis via activation of LKB-1/AMPK/Akt pathway and suppressing the activity of GSK-3β and p38α/MAPK [34]. Notably, cardio-myopathy may sometimes manifest hypertrophy of the heart muscle, which may contribute to heart failure if not treated in time. Dong and colleagues discovered that AMPK can increase the ejection fraction (EF) and decrease the protein synthesis rate in myocardial cells in mice with myocardial hypertrophy [76]. Expression of SIRT2 levels were downregulated in hypertrophic hearts. SIRT2 knockout markedly exaggerates cardiac hypertrophy and fibrosis as well as decreases in cardiac ejection fraction and fractional shortening in aged mice. Conversely, cardiac-specific SIRT2 overexpression is able to protect the hearts against Ang II-induced cardiac hypertrophy and fibrosis and rescued cardiac function. Mechanistically, SIRT2 maintains the activity of AMPK in aged and AngII-induced hypertrophic hearts *in vivo* as well as in cardiomyocytes *in vitro* via LKB1 [77]. Further study found that AMPK inhibit the myocardial hypertrophy through the regulation of the myocardial energy metabolism via SIRT1 signaling pathway. These results support the ability of AMPK to suppress oxidative stress, reduce apoptosis and regulate energy metabolism against myocardiopathy.

### 4.3 Heart failure

Heart failure is the final stage in the development of heart disease and a dominant cause of morbidity and mortality in the developed world [78]. It commonly affects older population and with the extension of life expectancy and the improvement of medical and health conditions, the number of patients with heart failure is expected to increase [79]. Recently, the protective effect of AMPK in heart failure has been demonstrated [80, 81].

Myocardial injury resulting from any cause, such as myocardial infarction, cardiomyopathy, or inflammation, can eventually lead to heart failure [82]. Previous studies have demonstrated that AMPK can inhibit cardiac hypertrophy and prevent progression of heart failure by promoting autophagy [39-41]. On the contrary, multiple researches have suggested that overactive and dysregulated autophagy may lead to the onset and exacerbation of heart failure [83-85]. Li et al observed that AMPK activation can significantly blunt the aggravation of heart failure and restore the cardiac function in failing hearts [80]. In their study, heart failure was induced by pressure overload via descending aortic banding (AB). Compared to that in the saline control group, after AB surgery, 4 weeks of AICAR (AMPK activator) treatment significantly decreased autophagy in the failing hearts. It’s important to point out that in the hearts without AB surgery, AICAR markedly increased autophagy. The results suggest that AMPK activation could reduce autophagy in failing hearts but induce autophagy in non-failing hearts. Further studies have found that AMPK improves the cardiac function during the development of chronic heart failure by attenuating autophagy, potentially via mTORC2 activation and the downstream effects. Not only does AMPK regulate autophagy to increase heart function, but also improves the heart failure and heart function by reducing ER stress and ROS levels, mediating various intracellular physiological functions, delaying myocardial fibrosis, and reducing heart damage [81]. Taken together, some drugs such as metformin, statin, trimetazidine, resveratrol etc., have the function of activating AMPK, and may be used as an alternative drug for the treatment of heart failure in the future.

## 5. AMPK as a therapeutic target

In recent years, numerous *in vitro* and *in vivo* studies have demonstrated AMPK’s protective role in heart disease, such as myocardial ischemia. However, the clinical study of AMPK in CVD is rare. Thus, to further demonstrate whether AMPK signaling activators can be used in clinical practice, more clinical studies are necessary. Nowadays, some drugs such as metformin, statin, trimetazidine, resveratrol etc. have been reported as the AMPK activators to play a protective role in CVD in animal models. Whether they can be applied to human diseases is still a problem.

Targeting autophagy has been confirmed a novel therapeutic strategy in cardiac ischemia/reperfusion injury. Notably, inducing autophagy via AMPK/mTOR signaling is protective within 6 h after ischemic episode while it becomes harmful after 6 h [86]. This result indicates that controlling the exact time of autophagy counts and might be taken as a window period for AMPK-induced autophagy in MI. Li et al demonstrated that inducing protective autophagy and autophagy flux via activation of AMPK-ULK1 signaling can also protect against pressure overload-induced cardiac hypertrophy, which is independent of mTOR signaling[41]. In addition, the activation of AMPK by metformin stimulated JNK1-Bcl-2 signaling and the disruption of the Beclin1-Bcl-2 complex restores autophagy and protects against cardiac apoptosis in diabetic mice [39]. Taken together, the activation of mTOR, ULK1 and JNK1-Bcl-2 signaling by activating AMPK may be the potential targets against cardiac injury.

Aldehyde dehydrogenase 2 (ALDH2), an abundantly expressed protein in heart, has an important role in cardiac aldehyde metabolism and detoxification [87]. Compelling evidence has indicated that targeting ALDH2 may be a new strategy for MI/R cure. Ma and his colleagues discovered that ALDH2 lead to enhanced Akt and AMPK activation, inhibition of Foxo3, apoptosis, and mitochondrial dysfunction against cardiac acute ethanol toxicity [88, 89]. Further studies revealed that ALDH2 protects against MI/R injury possibly through restoration of autophagy by AMPK-mTOR and Akt-mTOR signaling [90]. They observed ALDH2 significantly promotes autophagy during ischemia, which is accompanied by AMPK activation and mTOR inhibition. Thus, ALDH2 as a drug may play a protective role in MI/R injury. AMPK plays an important role in the myocardial protection of ALDH2. Notably, Ren and colleagues found that Aging enhances ROS production and reduced mitochondrial membrane potential, the effects of which were accentuated by AMPK deficiency. Moreover, treatment of the AMPK activator metformin attenuates aging-induced cardiomyocyte contractile defects [4].

## 6. Problems and prospects

Previous studies have mainly focused on the roles that AMPK plays in CVD; however, the regulatory function of AMPK in aging-related CVD has recently attracted great attention. In this review, we 1) introduced the protective mechanisms of AMPK signaling in the myocardium; 2) described the role of AMPK in cardiac aging; and 3) highlighted the research progress regarding the roles of AMPK signaling in CVD, including myocardial ischemia, cardiomyopathy, and heart failure.

Although several lines of evidence have clarified the specific mechanisms of aberrant AMPK signaling in MI, the development of successful treatments that target AMPK remain at its infancy. More translational research and additional clinical trials are needed before patients can benefit from the regulation of the AMPK signaling pathway. Further investigations may focus on 1) identifying the relationship between AMPK concentrations and the incidence and progression of MI, 2) determining how to retain AMPK signaling at appropriate levels to protect against MI, 3) investigating whether the clinical application of therapeutics targeting AMPK has any suboptimum potency, and 4) further studies on other molecules upstream and downstream of AMPK, which may be potential therapeutic targets for MI in the future. Altogether, this review presents a comprehensive picture of the roles that AMPK plays in MI and indicates the potential of AMPK as a novel therapeutic target for aging-related CVD.

## References

[1] Alfaras I, Di Germanio C, Bernier M, Csiszar A, Ungvari Z, Lakatta EG, et al (2016). Pharmacological Strategies to Retard Cardiovascular Aging. Circ Res, 118:1626-1642. 27174954 10.1161/CIRCRESAHA.116.307475 PMC4894351

[2] Benjamin EJ, Muntner P, Alonso A, Bittencourt MS, Callaway CW, Carson AP, et al (2019). Heart Disease and Stroke Statistics-2019 Update: A Report From the American Heart Association. Circulation, 139:e56-e528. 30700139 10.1161/CIR.0000000000000659

[3] Bittner VA (2019). The New 2019 ACC/AHA Guideline on the Primary Prevention of Cardiovascular Disease. Circulation. 10.1161/CIRCULATIONAHA.119.040625 30879338

[4] Turdi S, Fan X, Li J, Zhao J, Huff AF, Du M, et al (2010). AMP-activated protein kinase deficiency exacerbates aging-induced myocardial contractile dysfunction. Aging Cell, 9:592-606. 20477759 10.1111/j.1474-9726.2010.00586.x PMC2910211

[5] McClellan M, Brown N, Califf RM, Warner JJ (2019). Call to Action: Urgent Challenges in Cardiovascular Disease: A Presidential Advisory From the American Heart Association. Circulation, 139:e44-e54. 30674212 10.1161/CIR.0000000000000652

[6] Kudo N, Barr AJ, Barr RL, Desai S, Lopaschuk GD (1995). High rates of fatty acid oxidation during reperfusion of ischemic hearts are associated with a decrease in malonyl-CoA levels due to an increase in 5'-AMP-activated protein kinase inhibition of acetyl-CoA carboxylase. J Biol Chem, 270:17513-17520. 7615556 10.1074/jbc.270.29.17513

[7] Sambandam N, Lopaschuk GD (2003). AMP-activated protein kinase (AMPK) control of fatty acid and glucose metabolism in the ischemic heart. Prog Lipid Res, 42:238-256. 12689619 10.1016/s0163-7827(02)00065-6

[8] Salt IP, Hardie DG (2017). AMP-Activated Protein Kinase: An Ubiquitous Signaling Pathway With Key Roles in the Cardiovascular System. Circ Res, 120:1825-1841. 28546359 10.1161/CIRCRESAHA.117.309633 PMC5447810

[9] Lu Q, Li X, Liu J, Sun X, Rousselle T, Ren D, et al (2019). AMPK is associated with the beneficial effects of antidiabetic agents on cardiovascular diseases. Biosci Rep, 39. 10.1042/BSR20181995 PMC6379227 30710062

[10] Feng Y, Lu Y, Liu D, Zhang W, Liu J, Tang H, et al (2018). Apigenin-7-O-beta-d-(-6''-p-coumaroyl)-glucopyranoside pretreatment attenuates myocardial ischemia/reperfusion injury via activating AMPK signaling. Life Sci, 203:246-254. 29705352 10.1016/j.lfs.2018.04.048

[11] Slamova K, Papousek F, Janovska P, Kopecky J, Kolar F (2016). Adverse effects of AMP-activated protein kinase alpha2-subunit deletion and high-fat diet on heart function and ischemic tolerance in aged female mice. Physiol Res, 65:33-42. 26596312 10.33549/physiolres.932979

[12] van der Pol A, van Gilst WH, Voors AA, van der Meer P (2019). Treating oxidative stress in heart failure: past, present and future. Eur J Heart Fail, 21:425-435. 30338885 10.1002/ejhf.1320 PMC6607515

[13] Munzel T, Camici GG, Maack C, Bonetti NR, Fuster V, Kovacic JC (2017). Impact of Oxidative Stress on the Heart and Vasculature: Part 2 of a 3-Part Series. J Am Coll Cardiol, 70:212-229. 28683969 10.1016/j.jacc.2017.05.035 PMC5663297

[14] Li X, Wu D, Tian Y (2018). Fibroblast growth factor 19 protects the heart from oxidative stress-induced diabetic cardiomyopathy via activation of AMPK/Nrf2/HO-1 pathway. Biochem Biophys Res Commun, 502:62-68. 29778534 10.1016/j.bbrc.2018.05.121

[15] Yang H, Feng A, Lin S, Yu L, Lin X, Yan X, et al (2018). Fibroblast growth factor-21 prevents diabetic cardiomyopathy via AMPK-mediated antioxidation and lipid-lowering effects in the heart. Cell Death Dis, 9:227. 29445083 10.1038/s41419-018-0307-5 PMC5833682

[16] Zhao C, Zhang Y, Liu H, Li P, Zhang H, Cheng G (2017). Fortunellin protects against high fructose-induced diabetic heart injury in mice by suppressing inflammation and oxidative stress via AMPK/Nrf-2 pathway regulation. Biochem Biophys Res Commun, 490:552-559. 28624452 10.1016/j.bbrc.2017.06.076

[17] Kosuru R, Cai Y, Kandula V, Yan D, Wang C, Zheng H, et al (2018). AMPK Contributes to Cardioprotective Effects of Pterostilbene Against Myocardial Ischemia- Reperfusion Injury in Diabetic Rats by Suppressing Cardiac Oxidative Stress and Apoptosis. Cell Physiol Biochem, 46:1381-1397. 29689567 10.1159/000489154

[18] Kosuru R, Kandula V, Rai U, Prakash S, Xia Z, Singh S (2018). Pterostilbene Decreases Cardiac Oxidative Stress and Inflammation via Activation of AMPK/Nrf2/HO-1 Pathway in Fructose-Fed Diabetic Rats. Cardiovasc Drugs Ther, 32:147-163. 29556862 10.1007/s10557-018-6780-3

[19] Guo S, Yao Q, Ke Z, Chen H, Wu J, Liu C (2015). Resveratrol attenuates high glucose-induced oxidative stress and cardiomyocyte apoptosis through AMPK. Mol Cell Endocrinol, 412:85-94. 26054749 10.1016/j.mce.2015.05.034

[20] Elmore S (2007). Apoptosis: a review of programmed cell death. Toxicol Pathol, 35:495-516. 17562483 10.1080/01926230701320337 PMC2117903

[21] Grilo AL, Mantalaris A (2019). Apoptosis: A mammalian cell bioprocessing perspective. Biotechnol Adv, 37:459-475. 30797096 10.1016/j.biotechadv.2019.02.012

[22] Erekat NS 2018 Apoptosis and its Role in Parkinson's Disease. In Parkinson's Disease: Pathogenesis and Clinical Aspects. Stoker TB, and Greenland JC, editors. Brisbane (AU).

[23] Arguelles S, Guerrero-Castilla A, Cano M, Munoz MF, Ayala A (2019). Advantages and disadvantages of apoptosis in the aging process. Ann N Y Acad Sci, 1443:20-33. 30839127 10.1111/nyas.14020

[24] Nakka VP, Gusain A, Mehta SL, Raghubir R (2008). Molecular mechanisms of apoptosis in cerebral ischemia: multiple neuroprotective opportunities. Mol Neurobiol, 37:7-38. 18066503 10.1007/s12035-007-8013-9

[25] Ichim G, Tait SW (2016). A fate worse than death: apoptosis as an oncogenic process. Nat Rev Cancer, 16:539-548. 27364482 10.1038/nrc.2016.58

[26] Zhang Z, Wang S, Zhou S, Yan X, Wang Y, Chen J, et al (2014). Sulforaphane prevents the development of cardiomyopathy in type 2 diabetic mice probably by reversing oxidative stress-induced inhibition of LKB1/AMPK pathway. J Mol Cell Cardiol, 77:42-52. 25268649 10.1016/j.yjmcc.2014.09.022

[27] Zhang L, Ding WY, Wang ZH, Tang MX, Wang F, Li Y, et al (2016). Early administration of trimetazidine attenuates diabetic cardiomyopathy in rats by alleviating fibrosis, reducing apoptosis and enhancing autophagy. J Transl Med, 14:109. 27121077 10.1186/s12967-016-0849-1 PMC4848862

[28] Yeh CH, Chen TP, Wang YC, Lin YM, Fang SW (2010). AMP-activated protein kinase activation during cardioplegia-induced hypoxia/reoxygenation injury attenuates cardiomyocytic apoptosis via reduction of endoplasmic reticulum stress. Mediators Inflamm, 2010:130636. 21318153 10.1155/2010/130636 PMC3034973

[29] Zhang GG, Cai HQ, Li YH, Sui YB, Zhang JS, Chang JR, et al (2013). Ghrelin protects heart against ERS-induced injury and apoptosis by activating AMP-activated protein kinase. Peptides, 48:156-165. 23994559 10.1016/j.peptides.2013.08.015

[30] Woehlbier U, Hetz C (2011). Modulating stress responses by the UPRosome: a matter of life and death. Trends Biochem Sci, 36:329-337. 21482118 10.1016/j.tibs.2011.03.001

[31] Nam DH, Han JH, Kim S, Shin Y, Lim JH, Choi HC, et al (2016). Activated protein C prevents methylglyoxal-induced endoplasmic reticulum stress and cardiomyocyte apoptosis via regulation of the AMP-activated protein kinase signaling pathway. Biochem Biophys Res Commun, 480:622-628. 27794481 10.1016/j.bbrc.2016.10.106

[32] Jung TW, Lee MW, Lee YJ, Kim SM (2012). Metformin prevents endoplasmic reticulum stress-induced apoptosis through AMPK-PI3K-c-Jun NH2 pathway. Biochem Biophys Res Commun, 417:147-152. 22138650 10.1016/j.bbrc.2011.11.073

[33] Liu D, Ma Z, Di S, Yang Y, Yang J, Xu L, et al (2018). AMPK/PGC1alpha activation by melatonin attenuates acute doxorubicin cardiotoxicity via alleviating mitochondrial oxidative damage and apoptosis. Free Radic Biol Med, 129:59-72. 30172748 10.1016/j.freeradbiomed.2018.08.032

[34] Al-Damry NT, Attia HA, Al-Rasheed NM, Al-Rasheed NM, Mohamad RA, Al-Amin MA, et al (2018). Sitagliptin attenuates myocardial apoptosis via activating LKB-1/AMPK/Akt pathway and suppressing the activity of GSK-3beta and p38alpha/MAPK in a rat model of diabetic cardiomyopathy. Biomed Pharmacother, 107:347-358. 30099338 10.1016/j.biopha.2018.07.126

[35] Moloudizargari M, Asghari MH, Ghobadi E, Fallah M, Rasouli S, Abdollahi M (2017). Autophagy, its mechanisms and regulation: Implications in neurodegenerative diseases. Ageing Res Rev, 40:64-74. 28923312 10.1016/j.arr.2017.09.005

[36] Mialet-Perez J, Vindis C (2017). Autophagy in health and disease: focus on the cardiovascular system. Essays Biochem, 61:721-732. 29233881 10.1042/EBC20170022

[37] Saito T, Kuma A, Sugiura Y, Ichimura Y, Obata M, Kitamura H, et al (2019). Autophagy regulates lipid metabolism through selective turnover of NCoR1. Nat Commun, 10:1567. 30952864 10.1038/s41467-019-08829-3 PMC6450892

[38] Zech ATL, Singh SR, Schlossarek S, Carrier L (2019). Autophagy in cardiomyopathies. Biochim Biophys Acta Mol Cell Res. 10.1016/j.bbamcr.2019.01.013 30831130

[39] He C, Zhu H, Li H, Zou MH, Xie Z (2013). Dissociation of Bcl-2-Beclin1 complex by activated AMPK enhances cardiac autophagy and protects against cardiomyocyte apoptosis in diabetes. Diabetes, 62:1270-1281. 23223177 10.2337/db12-0533 PMC3609561

[40] Zou MH, Xie Z (2013). Regulation of interplay between autophagy and apoptosis in the diabetic heart: new role of AMPK. Autophagy, 9:624-625. 23380689 10.4161/auto.23577 PMC3627682

[41] Li RL, Wu SS, Wu Y, Wang XX, Chen HY, Xin JJ, et al (2018). Irisin alleviates pressure overload-induced cardiac hypertrophy by inducing protective autophagy via mTOR-independent activation of the AMPK-ULK1 pathway. J Mol Cell Cardiol, 121:242-255. 30053525 10.1016/j.yjmcc.2018.07.250

[42] Alers S, Loffler AS, Wesselborg S, Stork B (2012). Role of AMPK-mTOR-Ulk1/2 in the regulation of autophagy: cross talk, shortcuts, and feedbacks. Mol Cell Biol, 32:2-11. 22025673 10.1128/MCB.06159-11 PMC3255710

[43] Takagi H, Matsui Y, Hirotani S, Sakoda H, Asano T, Sadoshima J (2007). AMPK mediates autophagy during myocardial ischemia in vivo. Autophagy, 3:405-407. 17471015 10.4161/auto.4281

[44] Matsui Y, Takagi H, Qu X, Abdellatif M, Sakoda H, Asano T, et al (2007). Distinct roles of autophagy in the heart during ischemia and reperfusion: roles of AMP-activated protein kinase and Beclin 1 in mediating autophagy. Circ Res, 100:914-922. 17332429 10.1161/01.RES.0000261924.76669.36

[45] Wang Y, Yang Z, Zheng G, Yu L, Yin Y, Mu N, et al (2019). Metformin promotes autophagy in ischemia/reperfusion myocardium via cytoplasmic AMPKalpha1 and nuclear AMPKalpha2 pathways. Life Sci, 225:64-71. 30953640 10.1016/j.lfs.2019.04.002

[46] Li C, Yu L, Xue H, Yang Z, Yin Y, Zhang B, et al (2017). Nuclear AMPK regulated CARM1 stabilization impacts autophagy in aged heart. Biochem Biophys Res Commun, 486:398-405. 28315332 10.1016/j.bbrc.2017.03.053

[47] Wang S, Kandadi MR, Ren J (2019). Double knockout of Akt2 and AMPK predisposes cardiac aging without affecting lifespan: Role of autophagy and mitophagy. Biochim Biophys Acta Mol Basis Dis, 1865:1865-1875. 31109453 10.1016/j.bbadis.2018.08.011 PMC6530587

[48] Ren J, Yang L, Zhu L, Xu X, Ceylan AF, Guo W, et al (2017). Akt2 ablation prolongs life span and improves myocardial contractile function with adaptive cardiac remodeling: role of Sirt1-mediated autophagy regulation. Aging Cell, 16:976-987. 28681509 10.1111/acel.12616 PMC5595687

[49] Hua Y, Zhang Y, Ceylan-Isik AF, Wold LE, Nunn JM, Ren J (2011). Chronic Akt activation accentuates aging-induced cardiac hypertrophy and myocardial contractile dysfunction: role of autophagy. Basic Res Cardiol, 106:1173-1191. 21901288 10.1007/s00395-011-0222-8

[50] Zhang Y, Wang C, Zhou J, Sun A, Hueckstaedt LK, Ge J, et al (2017). Complex inhibition of autophagy by mitochondrial aldehyde dehydrogenase shortens lifespan and exacerbates cardiac aging. Biochim Biophys Acta Mol Basis Dis, 1863:1919-1932. 28347844 10.1016/j.bbadis.2017.03.016

[51] Medzhitov R (2008). Origin and physiological roles of inflammation. Nature, 454:428-435. 18650913 10.1038/nature07201

[52] Kuprash DV, Nedospasov SA (2016). Molecular and Cellular Mechanisms of Inflammation. Biochemistry (Mosc), 81:1237-1239. 27914449 10.1134/S0006297916110018

[53] Wellen KE, Hotamisligil GS (2005). Inflammation, stress, and diabetes. J Clin Invest, 115:1111-1119. 15864338 10.1172/JCI25102 PMC1087185

[54] Chen X, Li X, Zhang W, He J, Xu B, Lei B, et al (2018). Activation of AMPK inhibits inflammatory response during hypoxia and reoxygenation through modulating JNK-mediated NF-kappaB pathway. Metabolism, 83:256-270. 29526538 10.1016/j.metabol.2018.03.004 PMC5960613

[55] Wang X, Guo Z, Ding Z, Mehta JL (2018). Inflammation, Autophagy, and Apoptosis After Myocardial Infarction. J Am Heart Assoc, 7. 10.1161/JAHA.117.008024 PMC6015297 29680826

[56] Ren J, Xu X, Wang Q, Ren SY, Dong M, Zhang Y (2016). Permissive role of AMPK and autophagy in adiponectin deficiency-accentuated myocardial injury and inflammation in endotoxemia. J Mol Cell Cardiol, 93:18-31. 26906634 10.1016/j.yjmcc.2016.02.002

[57] Cieslik KA, Trial J, Entman ML (2017). Aicar treatment reduces interstitial fibrosis in aging mice: Suppression of the inflammatory fibroblast. J Mol Cell Cardiol, 111:81-85. 28826664 10.1016/j.yjmcc.2017.08.003 PMC5600709

[58] Xu X, Pang J, Chen Y, Bucala R, Zhang Y, Ren J (2016). Macrophage Migration Inhibitory Factor (MIF) Deficiency Exacerbates Aging-Induced Cardiac Remodeling and Dysfunction Despite Improved Inflammation: Role of Autophagy Regulation. Sci Rep, 6:22488. 26940544 10.1038/srep22488 PMC4778027

[59] Greco S, Gorospe M, Martelli F (2015). Noncoding RNA in age-related cardiovascular diseases. J Mol Cell Cardiol, 83:142-155. 25640162 10.1016/j.yjmcc.2015.01.011 PMC5509469

[60] Picca A, Mankowski RT, Burman JL, Donisi L, Kim JS, Marzetti E, et al (2018). Mitochondrial quality control mechanisms as molecular targets in cardiac ageing. Nat Rev Cardiol, 15:543-554. 30042431 10.1038/s41569-018-0059-z PMC6283278

[61] Gude NA, Broughton KM, Firouzi F, Sussman MA (2018). Cardiac ageing: extrinsic and intrinsic factors in cellular renewal and senescence. Nat Rev Cardiol, 15:523-542. 30054574 10.1038/s41569-018-0061-5

[62] Costantino S, Paneni F, Cosentino F (2016). Ageing, metabolism and cardiovascular disease. J Physiol, 594:2061-2073. 26391109 10.1113/JP270538 PMC4933114

[63] Ding YN, Tang X, Chen HZ, Liu DP (2018). Epigenetic Regulation of Vascular Aging and Age-Related Vascular Diseases. Adv Exp Med Biol, 1086:55-75. 30232752 10.1007/978-981-13-1117-8_4

[64] Ma H, Wang J, Thomas DP, Tong C, Leng L, Wang W, et al (2010). Impaired macrophage migration inhibitory factor-AMP-activated protein kinase activation and ischemic recovery in the senescent heart. Circulation, 122:282-292. 20606117 10.1161/CIRCULATIONAHA.110.953208 PMC2907453

[65] Chen Q, Lesnefsky EJ (2018). A new strategy to decrease cardiac injury in aged heart following ischaemia-reperfusion: enhancement of the interaction between AMPK and SIRT1. Cardiovasc Res, 114:771-772. 29596586 10.1093/cvr/cvy062 PMC5909644

[66] Abdellatif M, Sedej S, Carmona-Gutierrez D, Madeo F, Kroemer G (2018). Autophagy in Cardiovascular Aging. Circ Res, 123:803-824. 30355077 10.1161/CIRCRESAHA.118.312208

[67] Favero G, Franceschetti L, Buffoli B, Moghadasian MH, Reiter RJ, Rodella LF, et al (2017). Melatonin: Protection against age-related cardiac pathology. Ageing Res Rev, 35:336-349. 27884595 10.1016/j.arr.2016.11.007

[68] Kataoka Y, Shibata R, Ohashi K, Kambara T, Enomoto T, Uemura Y, et al (2014). Omentin prevents myocardial ischemic injury through AMP-activated protein kinase- and Akt-dependent mechanisms. J Am Coll Cardiol, 63:2722-2733. 24768874 10.1016/j.jacc.2014.03.032

[69] Liu Z, Chen JM, Huang H, Kuznicki M, Zheng S, Sun W, et al (2016). The protective effect of trimetazidine on myocardial ischemia/reperfusion injury through activating AMPK and ERK signaling pathway. Metabolism, 65:122-130. 26892523 10.1016/j.metabol.2015.10.022 PMC4967934

[70] Tian L, Cao W, Yue R, Yuan Y, Guo X, Qin D, et al (2019). Pretreatment with Tilianin improves mitochondrial energy metabolism and oxidative stress in rats with myocardial ischemia/reperfusion injury via AMPK/SIRT1/PGC-1 alpha signaling pathway. J Pharmacol Sci, 139:352-360. 30910451 10.1016/j.jphs.2019.02.008

[71] Zhang Y, Wang Y, Xu J, Tian F, Hu S, Chen Y, et al (2019). Melatonin attenuates myocardial ischemia-reperfusion injury via improving mitochondrial fusion/mitophagy and activating the AMPK-OPA1 signaling pathways. J Pineal Res, 66:e12542. 30516280 10.1111/jpi.12542

[72] Lee JH, Budanov AV, Park EJ, Birse R, Kim TE, Perkins GA, et al (2010). Sestrin as a feedback inhibitor of TOR that prevents age-related pathologies. Science, 327:1223-1228. 20203043 10.1126/science.1182228 PMC2866632

[73] Quan N, Sun W, Wang L, Chen X, Bogan JS, Zhou X, et al (2017). Sestrin2 prevents age-related intolerance to ischemia and reperfusion injury by modulating substrate metabolism. FASEB J, 31:4153-4167. 28592638 10.1096/fj.201700063R PMC5572689

[74] Liu Y, Zhao YB, Wang SW, Zhou Y, Tang ZS, Li F (2017). Mulberry granules protect against diabetic cardiomyopathy through the AMPK/Nrf2 pathway. Int J Mol Med, 40:913-921. 28677741 10.3892/ijmm.2017.3050

[75] Sung MM, Zordoky BN, Bujak AL, Lally JS, Fung D, Young ME, et al (2015). AMPK deficiency in cardiac muscle results in dilated cardiomyopathy in the absence of changes in energy metabolism. Cardiovasc Res, 107:235-245. 26023060 10.1093/cvr/cvv166 PMC4565988

[76] Dong HW, Zhang LF, Bao SL (2018). AMPK regulates energy metabolism through the SIRT1 signaling pathway to improve myocardial hypertrophy. Eur Rev Med Pharmacol Sci, 22:2757-2766. 29771428 10.26355/eurrev_201805_14973

[77] Tang X, Chen XF, Wang NY, Wang XM, Liang ST, Zheng W, et al (2017). SIRT2 Acts as a Cardioprotective Deacetylase in Pathological Cardiac Hypertrophy. Circulation, 136:2051-2067. 28947430 10.1161/CIRCULATIONAHA.117.028728 PMC5698109

[78] Alexanian M, Padmanabhan A, McKinsey TA, Haldar SM (2019). Epigenetic therapies in heart failure. J Mol Cell Cardiol, 130:197-204. 30991033 10.1016/j.yjmcc.2019.04.012 PMC6850768

[79] Gedela M, Khan M, Jonsson O (2015). Heart Failure. S D Med, 68:403-405, 407-409. 26489162

[80] Li Y, Wang Y, Zou M, Chen C, Chen Y, Xue R, et al (2018). AMPK blunts chronic heart failure by inhibiting autophagy. Biosci Rep, 38. 10.1042/BSR20170982 PMC6050195 30021848

[81] Li X, Liu J, Lu Q, Ren D, Sun X, Rousselle T, et al (2019). AMPK: a therapeutic target of heart failure-not only metabolism regulation. Biosci Rep, 39. 10.1042/BSR20181767 PMC6328861 30514824

[82] Tanai E, Frantz S (2015). Pathophysiology of Heart Failure. Compr Physiol, 6:187-214. 26756631 10.1002/cphy.c140055

[83] Zhu H, Tannous P, Johnstone JL, Kong Y, Shelton JM, Richardson JA, et al (2007). Cardiac autophagy is a maladaptive response to hemodynamic stress. J Clin Invest, 117:1782-1793. 17607355 10.1172/JCI27523 PMC1890995

[84] Kostin S, Pool L, Elsasser A, Hein S, Drexler HC, Arnon E, et al (2003). Myocytes die by multiple mechanisms in failing human hearts. Circ Res, 92:715-724. 12649263 10.1161/01.RES.0000067471.95890.5C

[85] Hein S, Arnon E, Kostin S, Schonburg M, Elsasser A, Polyakova V, et al (2003). Progression from compensated hypertrophy to failure in the pressure-overloaded human heart: structural deterioration and compensatory mechanisms. Circulation, 107:984-991. 12600911 10.1161/01.cir.0000051865.66123.b7

[86] Davidson AJ, Disma N, de Graaff JC, Withington DE, Dorris L, Bell G, et al (2016). Neurodevelopmental outcome at 2 years of age after general anaesthesia and awake-regional anaesthesia in infancy (GAS): an international multicentre, randomised controlled trial. Lancet, 387:239-250. 26507180 10.1016/S0140-6736(15)00608-X PMC5023520

[87] Li C, Sun W, Gu C, Yang Z, Quan N, Yang J, et al (2018). Targeting ALDH2 for Therapeutic Interventions in Chronic Pain-Related Myocardial Ischemic Susceptibility. Theranostics, 8:1027-1041. 29463997 10.7150/thno.22414 PMC5817108

[88] Ma H, Li J, Gao F, Ren J (2009). Aldehyde dehydrogenase 2 ameliorates acute cardiac toxicity of ethanol: role of protein phosphatase and forkhead transcription factor. J Am Coll Cardiol, 54:2187-2196. 19942091 10.1016/j.jacc.2009.04.100

[89] Ma H, Yu L, Byra EA, Hu N, Kitagawa K, Nakayama KI, et al (2010). Aldehyde dehydrogenase 2 knockout accentuates ethanol-induced cardiac depression: role of protein phosphatases. J Mol Cell Cardiol, 49:322-329. 20362583 10.1016/j.yjmcc.2010.03.017 PMC2885537

[90] Ma H, Guo R, Yu L, Zhang Y, Ren J (2011). Aldehyde dehydrogenase 2 (ALDH2) rescues myocardial ischaemia/reperfusion injury: role of autophagy paradox and toxic aldehyde. Eur Heart J, 32:1025-1038. 20705694 10.1093/eurheartj/ehq253 PMC3076664

